# Preference of elderly patients’ to oral or intravenous chemotherapy in heavily pre-treated recurrent ovarian cancer: final results of a prospective multicenter trial

**DOI:** 10.1186/s40661-017-0040-2

**Published:** 2017-03-07

**Authors:** Radoslav Chekerov, Philipp Harter, Stefan Fuxius, Lars Christian Hanker, Linn Woelber, Lothar Müller, Peter Klare, Wolfgang Abenhardt, Yoana Nedkova, Isil Yalcinkaya, Georg Heinrich, Harald Sommer, Sven Mahner, Pauline Wimberger, Dominique Koensgen-Mustea, Rolf Richter, Gülten Oskay-Oezcelik, Jalid Sehouli, On behalf of the Ovarian Cancer Study Group of the North-Eastern German Society of Gynaecological Oncology (NOGGO)

**Affiliations:** 1 Department of Gynaecology, European Competence Centre for Ovarian Cancer, Charité Universitätsmedizin, Berlin, Germany; 2 Department of Gynaecology and Gynaecologic Oncology, Kliniken Essen Mitte, Essen, Germany; 3 Onkologische Schwerpunktpraxis Heidelberg, Heidelberg, Germany; 4 Department of Gynaecology and Obstetrics, Gynaecological Cancer Center of the University Schleswig-Holstein, Campus Lübeck, Lübeck, Germany; 5 Department of Gynaecology and Gynaecologic Oncology, University Medical Center Hamburg-Eppendorf, Hamburg, Germany; 6 Onkologische Schwerpunktpraxis Leer, Leer, Germany; 7 Praxisklinik Krebsheilkunde für Frauen, Berlin, Germany; 8 Onkologische Schwerpunktpraxis im Elisenhof, Munich, Germany; 9 Gynäkologisch-onkologische Schwerpunktpraxis, Fürstenwalde, Germany; 10 Department of Gynaecology and Obstetrics and Comprehensive Cancer Center, University of Munich, Munich, Germany; 11 Department of Gynecology and Obstetrics, Technische Universität Dresden, Dresden, Germany; 12 Department of Gynaecology and Obstetrics, University Medicine Greifswald, Greifswald, Germany; 13 North-Eastern German Society for Gynaecologic Oncology, Ovarian Cancer Study Group, Berlin, Germany

**Keywords:** Recurrent ovarian cancer, Elderly, Patient preference, Treosulfan

## Abstract

**Background:**

Palliative systemic treatment in elderly gynaecological cancer patients remains a major challenge. In recurrent ovarian cancer (ROC), treosulfan an active alkylating drug showed similar cytotoxicity whether as oral (p.o.) or intravenous (i.v.) application. The aim of this innovative trial was to evaluate the preference of elderly patients (≥65 years) for p.o. or i.v. chemotherapy focusing compliance, outcome, toxicities, and geriatric aspects as secondary endpoints.

**Methods:**

Patients with ROC had the free choice between treosulfan i.v. (7000 mg/m^2^ d1, q29d) or p.o. (600 mg/m^2^ daily d1-28, q57d). Only indecisive participants were randomized.

**Results:**

Overall 123 patients with 2^nd^ to 5^th^ recurrence were registered and 119 received at least one cycle of chemotherapy. 85.7% preferred treosulfan i.v. and 14.3% oral, where only three patients were randomized. Main reasons for i.v. preference associated with individual expectations of lower rate of gastrointestinal disorders, higher activity and tolerability of treatment. Median of applied chemotherapies was three (range 1–12 cycles), with most common grade 3/4 toxicities thrombopenia (18.7%), leukopenia (15.7%), ascites (7.6%), bowel obstruction (6.7%), and abdominal pain (4.2%). Median time until progression/overall survival was 5.2/7.8 months (i.v.), and 5.6/10.4 months (p.o.), respectively, without significant differences in efficacy.

**Conclusions:**

Elderly patients with recurrent ovarian cancer asked and demonstrated active participation in the decision-making process of their oncological treatment and favoured predominantly the i.v. application. Treosulfan was generally well-tolerated despite comorbidities and heavy pre-treatment. Our study demonstrates that patients’ preference did not influence prognosis negatively and remains important in gynaecologic oncology decision practice.

**EudraCT Nr.:**

2004-000719-25; NCT 00170690

## Background

Treatment of elderly ovarian cancer patients remain a great challenge in the palliative situation, where innovative therapies conflicts with clinical routine [1–3]. In general some physicians consider critical surgical or systemic treatment [4, 5] while many observers reported inadequate treatment quality in elderly compared to younger patients [3, 6–8]. Otherwise age was one of the common exclusion criteria in clinical trials, so their results could not consequently be transferred to senior cancer cohorts. Additional, age-dependent limitations of functional reserves are not well understood, but complex and require elaborate assessment [9, 10]. In particular, individual preferences and knowledge of patient reported outcome measures are key aspects of palliative concepts [11]. Resent published data have confirmed opposing expectations and individual preferences by cancer patients and their physicians [11–13]. Today data focusing elderly ovarian cancer patients, their preferences and expectations for therapy are still limited, thus to change the primary perspective in a clinical trial is provoking but could generate a helpful insight to gynaecologic oncologists.

As factors of decision-making in oncology are poorly understood, there is an ongoing intensive discussion about new conceptual and scientific approaches [14]. Not only efficacy and toxicity, but also patient’s acceptance of and compliance with treatment can significantly influence outcome [1, 3]. Inadequate therapy of elderly results quite often due to the erroneous belief that age alone determines lower tolerability to surgery and chemotherapy [15]. There is also controversial experience that, even in a palliation, elderly women can possibly tolerate debulking surgery and chemotherapy well, but still prediction of the individual aspects, benefits and risk is still not possible [8, 16]. Thus optimising strategies for increasing compliance and satisfaction with care should involve patients into the treatment decision-making process, above all respecting their expectations and preferences [13, 17, 18].

Treosulfan is a bifunctional alkylating prodrug showing activity for the i.v. formulation either as a single agent or in combination with other cytotoxic drugs such a cisplatin [19–21]. Furthermore, oral treosulfan demonstrated a high and constant bioavailability [22], which may lead to the same efficacy. Since both formulations show a similar efficacy and moderate toxicity, it seems attractive for evaluating individual therapy preferences. Treosulfan is approved in several European countries for the treatment of ovarian cancer and characterized by proven effectivity and mild toxicity (e.g. little hair loss and non-haematological side-effects), which makes it attractive for geriatric and multimorbid patients [23].

The innovative concept of this trial involved elderly patients with recurrent ovarian cancer to determine active their preference for therapy after detailed patient consultation on treatment aims and risks. Primary study objectives were the individual preference and free patient’s choice to chemotherapy with either i.v. or p.o. treosulfan. Additionally, we evaluated the reasons for individual choice and analysed compliance, tolerability, and efficacy of the different application routes. Patients were free to participate on geriatric assessment measures.

## Methods

### Study design

This was an open-label, multicentre trial of treosulfan in elderly women with ROC. Patients were enrolled into the study after failure of platinum-containing treatment, irrespective of their treatment-free interval, following an innovative registration design: they were free to choose between oral and i.v. treosulfan treatment. Only indecisive participants were randomized.

Patients were enrolled at 27 German institutions (18 hospitals, nine outpatient facilities). Women ≥ 65 years with recurrent ovarian, peritoneal, or fallopian tube cancer were eligible. Key inclusion criteria were as follows (selection): ECOG ≤ 2, serum creatinine ≤ 1.25 × upper normal limit (UNL), bilirubin ≤ 1.25 × UNL (in the presence of liver metastases ≤ 5 × UNL), and adequate bone marrow function (leucocytes ≥ 2.0 · × 10^9^/l, and platelet count ≥ 100 × 10^9^/l). Initially, only patients in the second line situation (first recurrence) were allowed to participate, but due to emerging trial results and improvement of national guidelines for the treatment of ovarian cancer, the subsequent change of inclusion criteria to patients with at least two previous therapies (≥3^rd^ line situation) was amended.

The primary aim was to explore the preference and compliance of elderly participants for the palliative treatment with oral or i.v. medication. Secondary objectives included compliance, toxicity, progression-free and overall survival. Additional quality of life, functional and comorbidity measures and geriatric assessments were performed according to the preference of the participants.

This trial was planned by the North-Eastern German Society of Gynaecological Oncology (NOGGO) Ovarian Cancer Study Group. The study was performed according to ICH-GCP (International Conference on Harmonization - Good Clinical Practice) guidelines after obtaining central ethical committee’s approval and trial registration (EudraCT Nr.: 2004-000719-25; NCT 00170690). Written informed consent was provided by each participant.

### Treatment plan and toxicity evaluation

Patients received a standard dose of 7000 mg/m^2^ treosulfan i.v. on day 1 of a 28-day cycle or 600 mg/m^2^ p.o. on days 1–28 of a 56-day cycle for a maximum of 12 months or until disease-progression or development of unacceptable toxicity.

The screening started within 14 days prior to start of therapy included an evaluation of the medical history, a physical examination, and a tumour evaluation, staged by CA 125 and radiological imaging (chest x-ray, ultrasound, CT or MRI scan). Laboratory analyses comprised haematology (biweekly), serum chemistry, and urine analysis. Evaluation of response was performed every 12 weeks or in case of symptoms or signs of tumour progression.

Toxicity was classified according to the NCI- CTCAE version 2.0. Safety analyses were performed on all patients who received at least one therapy cycle. In order to account for the limited haematopoietic resources of elderly patients, chemotherapy was applied only if leukocyte was ≥ 3.5 × 10^9^/l and platelets ≥ 100× 10^9^/l.

In cases of dose reduction due to severe haematological toxicity, no re-escalation was allowed. Tumour progression, intolerable toxicity (grade 3/4), and/or a treatment delay > 2 weeks led to discontinuation of treatment.

### Statistical analysis

The preference for oral or i.v. treosulfan was expected to result in a variable compliance. Description of compliance differences between the two treatment arms by 15% was defined as clinically significant. We used Fleiss statistical measurement to optimize the sample size [24]. Setting the test criteria to alpha = 5%, beta = 20% and a drop-out rate of 5%, 160 patients were initially intended to be recruited for this trial. Due to a slow recruitment we performed a prior evaluation with 123 patients identifying highly representative differences in preference and compliance, which were statistically significant to close early trial recruitment.

Results are presented as proportions, means, medians, and rates, and their adequate measures of distribution. We used a one-sample test of proportions to address the primary hypothesis. All other endpoints were evaluated in an exploratory fashion, and 95% confidence intervals (CI) were computed where appropriate.

Evaluation of response was performed by CA 125 monthly and by radiological assessment every 3 months. Response was measured according to UICC-criteria and CA-125 assessment criteria, established by Rustin et al. [25]. Progression-free survival and overall survival were defined as the interval between the first day of the study drug application and disease progression or death due to any cause. Both were calculated by the Kaplan-Meier method.

## Results

### Patient characteristics

**Fig. 1:**
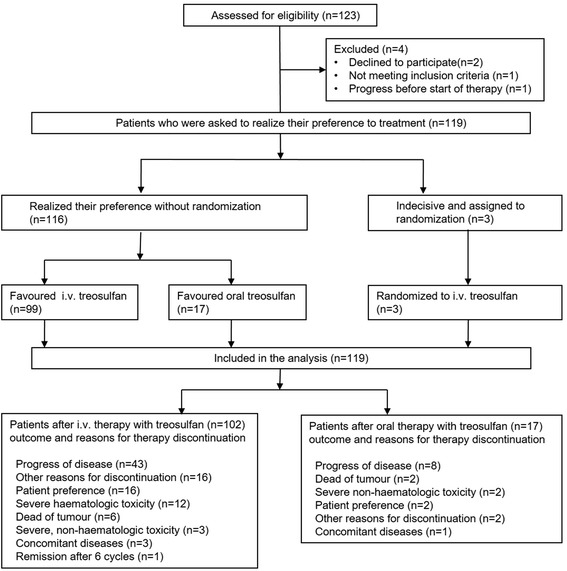
CONSORT diagram of trial profile

Out of 123 registered patients, 119 received at least one cycle of chemotherapy and were eligible for the final analysis (Fig. 1). Generally, there were no significant differences in the global patient characteristics (Table 1). The median age at recruitment was 71 years (range of 65–87 years). Most women were diagnosed with advanced stage III/IV high-grade carcinomas of serous-papillary histology and were in good condition (ECOG 0–1). The majority had received three previous cytotoxic treatments (three in the i.v. and two in the oral preference arm). Most patients were treated in this study due to second or third recurrence (56.3%), but 32% had four or more recurrences in their medical history. Because the protocol allowed to register patients independently of their platinum-free interval, the rate of late recurrences with a treatment-free interval >12 months was between 35 and 52% (oral vs. i.v. group). Distant metastases were rare and typically localized to the liver or lung. The median number of concomitant diseases was 5 (range 1–9), mostly of cardiovascular, musculoskeletal or gastrointestinal character (Table 2).

**Table 1:** Patients’ characteristics and distribution of clinical parameters according to individual preference, *n* = 119

Parameter of disease (n, %)	Preference to treatment	
	i.v. (*n* = 102)	oral (*n* = 17)
Median age, in years (range)	72 (65–87)	70 (65–77)
ECOG		
0	22 (21.6)	4 (23.5)
1	68 (66.7)	10 (58.8)
2	12 (11.7)	3 (17.7)
FIGO stage at primary diagnosis		
I	2 (2)	1 (5.9)
II	7 (6.9)	1 (5.9)
III	62 (60.8)	13 (76.5)
IV	25 (24.5)	2 (11.7)
not documented	6 (5.9)	-
Histology		
Serous papillary	59 (57.8)	13 (76.5)
Mucinous	12 (11.7)	3 (17.7)
Endometrioid	8 (7.8)	1 (5.9)
Others or NOS	23 (22.6)	-
Grading at primary diagnosis		
G1	2 (2)	-
G2	29 (28.4)	5 (29.4)
G3	61 (59.8)	12 (70.6)
not documented	10 (9.8)	-
Type of treatment in the adjuvant situation or last recurrence		
Surgical tumordebulking	100 (98)	17 (100)
Chemotherapy	101 (99)	17 (100)
Previous hormonal treatment	8 (7.8)	2 (11.8)
Previous Radiotherapy	6 (5.9)	1 (5.9)
Relapse-free interval after primary platinum based therapy		
< 6 months	21 (20.6)	4 (23.5)
6–12 months	28 (27.5)	7 (41.2)
> 12 months	53 (51.9)	6 (35.3)
Type of previous chemotherapy regimens (*n* = 372)		
platinum/taxan based	182 (56.9)	22 (44.9)
anthracyclin	38 (11.9)	6 (12.2)
topotecan	45 (14)	9 (18.4)
taxan	12 (3.8)	1 (2.1)
others	43 (13.4)	11 (22.4)
No. of previous chemotherapies for all median (min. / max.)	3 (1–8)	
	3 (1–8)	2 (1–7)
Recurrent situation at time of registration		
1. Recurrence	13 (12.7)	1 (5.9)
2. Recurrence	25 (24.5)	8 (47)
3. Recurrence	30 (29.4)	4 (23.5)
4. Recurrence	15 (14.7)	2 (11.8)
> 4 Recurrencies	19 (18.6)	2 (11.8)

**Table 2:** Reasons for treatment preference and concomitant diseases (*n* = 119)

Characteristics	i.v., *n* = 102, (%)	Oral, *n* = 17, (%)
Preference to therapy regime	99 (83.2)	17 (14.3)
Randomization (for indecisive patients)	3 (2.5)	0
Main reasons for therapy preference		
Wish to avoid gastrointestinal disorders	20 (19.6)	0
Disfavour / poor toleration of oral drugs	12 (17.8)	0
Oblivion / daily oral intake is unsure	14 (13.7)	0
Believe i.v. application is saver over i.v. port	15 (14.7)	0
More effective / higher treatment pressure	13 (12.3)	1 (5.9)
Oral drug application not possible - short bowel/subileus	4 (3.9)	0
Pre-existing chronic diarrhoea / vomiting	4 (3.9)	0
Expect better tolerability	4 (3.9)	0
Wish no hospital treatment / more independence / privacy	0	6 (35.3)
The handling of the therapy is simple	0	4 (23.5)
Continuity of the drug administration / maintenance effect	0	2 (11.8)
Made bad experience with venous puncture	0	1 (5.9)
Reason for preference not documented	16 (17.6)	3 (17.7)
Concomitant diseases (multiple answers)		
Cardiovascular	92 (90.2)	17 (100)
Musculoskeletal	37 (36.3)	7 (41.2)
Pulmonary	28 (27.5)	2 (11.8)
Lower gastrointestinal tract	39 (38.2)	4 (23.5)
Upper gastrointestinal tract	27 (26.5)	4 (23.5)
Metabolic and hormonal	25 (24.5)	6 (35.3)
Hepatic	27 (26.5)	3 (17.7)
Renal	12 (11.8)	6 (35.3)
Urinary tract	21 (20.6)	2 (11.8)
Neurological	27 (26.5)	7 (41.2)
Psychiatric	6 (5.9)	4 (23.5)

### Preference for chemotherapy

During the registration process patients were asked to realize their preference or to be randomized to treatment. Most them (*n* = 116, 97.5%) preferred to choose the application form of chemotherapy, thus only 3 indecisive women were randomized. A total of 85.7% or 102 patients realized their free choice to receive chemotherapy as i.v. application, where three were randomized to this arm. Seventeen patients (14.3%) preferred the oral therapy (no randomization to oral therapy). The main reasons for individual preference to i.v. or p.o. treosulfan are listed in Table 2.

### Toxicity profile

In both treatment arms, most non-haematological and haematological toxicities were of grade 1 or 2. The most common grade 3/4 haematological side-effects were thrombocytopenia, leukopenia, and neutropenia. Severe non-haematological events were rare. Moreover, a remarkably low rate of alopecia was observed (13.7% with grade 1/2, no grade 3). No therapy-related death was observed (Table 3).

**Table 3:** Toxicity, dose reduction and reasons for therapy discontinuation (*n* = 119)

Parameter	i.v., *n* = 102 (%)	Oral, *n* = 17 (%)
Toxicity (grade 3 or 4)	742 AE’s ^a^	14 AE’s
Haematological (all grade)		
Thrombocytopenia	38.6	30
Leucopenia	27.3	50
Neutropenia	16.3	10
Anemia	11.6	10
Febrile Neutropenia	7	-
Non-haematological (all grade)		
Ascites	9.9	11
Subileus (severe constipation)	8.6	11
Constipation	6.2	-
Abdominal pain	4.9	11
Ileus (bowel obstruction)	4.9	-
Vomiting	4.9	-
Nausea	3.7	-
Diarrhoea	2.5	11
Rectal incontinence	2.5	-
Others (< 1%)	51.8	56
Dose reduction (27 of all 421 cycles)	6.4	
(26 of 376 i.v. cycles vs. 1 of 45 oral cycles)	6.9	2.2
Prolongation of treatment interval (> 14d)	25	4.4
Reasons for early therapy discontinuation		
Progressive disease	42	47.1
Patients preference	15.7	11.8
Other reasons	15.7	11.8
Haematological toxicity (grade 3/4)	11.8	-
Dead of tumour	5.9	11.8
Non-haematological toxicity (grade 3/4)	2.9	11.8
Concomitant disease	2.9	5.9
Complete remission	1	-
Main cause of death		
Tumour related	80.4	82.4
Others	7.8	5.9

^a^adverse events

### Treatment delay and discontinuation

In total, 421 cycles of treosulfan (median 3, range 1–12) were administered. Dose reductions were performed in 27 courses of treosulfan therapy (6.4%, 1 oral and 26 i.v. arm, see Table 3), whereas for 96 courses (22.8%), an interval prolongation was necessary. The main reason for dose reduction were haematological AEs and for treatment delay haematological toxicity and organisational reasons/preference.

Six Patients (35.3%) preferring oral application received three courses of therapy, but only three patients (17.6%) finished all planned 6 cycles (12 months). In the i.v. group, 26 patients (25.4%) received six courses, but only one received the maximum of 12 chemotherapies. Disease progression and patient’s choice were main reasons for discontinuing treatment. Subsequently 11.8% of the i.v. participants discontinued therapy due to haematological toxicities, whereas non-haematological events were twice as high in the oral group (Table 3).

### Response, survival, and follow-up

Seventy-four patients (65 i.v./ 9 p.o.) were considered assessable for radiological response. One patient showed complete response (1/0), 13 (11/2) partial remission (PR), 15 (14/1) stable disease (SD), 45 (39/6) progressive disease (PD), and 45 were not assessable for response (lost of follow-up). During the study follow-up period, 105 patients (88.2%) died, mostly documented to disease progress.

**Fig. 2:**
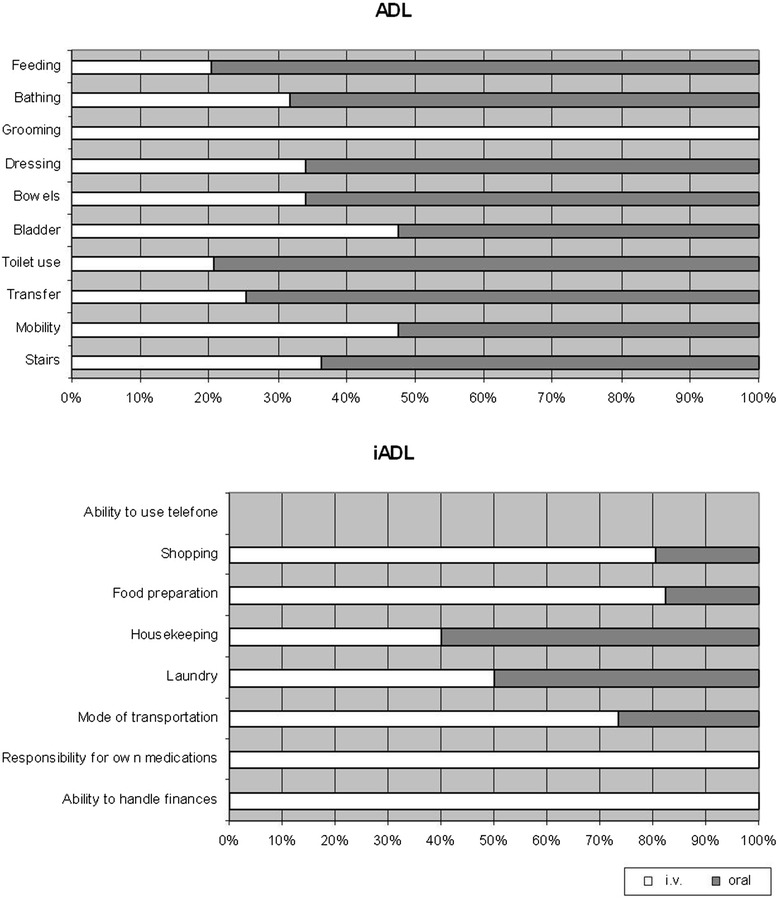
Distribution of severe geriatric assessment inside patients with i.v. and oral preference. ADL = activities of daily living; iADL = instrumental activities of daily living

Median follow-up was 11.4 months. Median progression-free survival in this study was 3.7 months (i.v. 3.5 months/p.o. 4.2 months). Median overall survival was 8.0 months, with 7.8 months (i.v.) and 10.4 months (p.o.), respectively (Fig. 2). There was no statistically significant difference between the two arms regarding survival.

### Geriatric aspects

The highest participation in the geriatric assessment with ADL and iADL questionnaires was achieved at the start of therapy (70%), but declined during the study period to less than 10%. Interestingly the proportion of patients which declared to need support or help in their activities of daily living (ADL) was significantly higher within the individuals with preference for oral treatment, but this effect was not demonstrated for the iADL-score. Geriatric measurements did not demonstrate specific differences in the patient preference profiles (Fig. 2).

## Discussion

The key objectives in the treatment of recurrent ovarian cancer (ROC) are preventing disease-related symptoms, prolonging progression-free survival, and maintaining quality of life [2, 26]. However, as more patients achieve long-term survival, palliative care has evolved to include all aspects of cancer survivorship, which increase the need of new and thoroughly considered approaches focusing individual expectations, preference and acceptance of treatment [13, 27, 28]. Demographic switch increase the expectations on clinicians and health care providers, since multimodal management and identification of subgroups with specific tumour characteristics is gaining key importance [3, 29]. In this prospective study, we evaluated the new strategy giving elderly patients the opportunity to choose free the application route of their chemotherapy and analysed prospectively their preferences. The great majority of 97% realized their preference, demonstrating clearly high motivation to participate to the decision making process. Unexpectedly most patients preferred the i.v. application of the drug, associating oral intake over long period with expected higher gastrointestinal risks for reflux, hyperacidemia, nausea, change of taste, loss of appetite or diarrhoea.

The monthly i.v. infusion seemed for many to be more comfortable, since regular hospital and physician contacts does not negatively influence patient’s autonomy and compliance, as described by others [5, 18]. These findings are remarkable, as physicians tend often to inconsequent management of geriatric patients [3, 5, 26]. Multiple analyses demonstrated in the past, that elderly were treated suboptimal, commonly under-represented in clinical trials, which resulted in their unfavourable outcome [3, 7]. Although data do not support the suggestion that age – independently of any other factors – is a negative prognostic factor, we need new clinical instruments to evaluate additionally aspects of acceptance, preference and satisfaction with care, as well as social and psychological scopes of treatment [1, 5]. Our trial offers here unique aspects and insights to traditional management and can help for more individualisation of palliative ovarian cancer care.

Patient’s preference is known to be complex, to base on individual experiences and reflecting on relevant life events and be difficult to assess [30, 31]. Acceptance of and compliance with oncological therapy plays a key role for improving efficacy and prolonging survival. Age is the strongest demographic factor affecting patients’ preferences: younger and better-educated patients, and women in general, were reported to prefer more active role in decision-making [31]. Degner et al. identified a large variation in preferred and attained levels of involvement in the treatment decisions for breast cancer patients [32]. Our analysis identified that, even after extensive pre-treatment (median of 3 previous therapies) and in highly palliative situation elderly ovarian cancer patients prefer to realize their individual preference and accept the corresponding treatment. Therapy discontinuation remained low, mostly due to tumour progression or toxicity. The i.v. regimen seems to demonstrate a partly favourable toxicity compared to oral treosulfan. Interestingly, this corresponds with the patients’ expectation of a milder effect on the gastrointestinal tract, which, in turn, was declared to guarantee more safety. On the other hand, patients expected a better control over their treatment since the application takes place in a hospital/outpatient department and associated the corresponding influence with expected positively affection on their outcome. This reflects report of close relationship of oncological patients to their treating physicians and palliative care teams as one of their most important representatives during the treatment [11, 13, 28]. Otherwise the possibility of realizing one’s own preference evidently has the strongest psychological impact [33].

Despite all limitations of the small patient cohort and the non-mandatory evaluation of geriatric measures, this trial report interesting insight into a very complex palliative cohort of geriatric patients. Although the observed disease stabilisation was mostly brief, only a small number of participants interrupted their study participation at their own request, keeping their compliance and acceptance high. Despite methodical limitations due to the imbalance of patient distribution, our results are comparable with data by Pfeiffer et al, who reported preference for i.v. chemotherapy within colon cancer outpatients, probably due to expected lower toxicity [18].. In contrast, other groups reported preference for oral chemotherapy, within younger breast cancer patients associated with their better functional and physiological status, less comorbidities, and the wish for more individuality in their daily activities [1, 13].. A certain bias in the trial design consisted, perhaps, in the fact that, in most cases, in these trials an established i.v. drug was compared to an “innovative” oral formulation, which seemed more attractive [17, 18, 32, 34].

The frequency of concomitant diseases registered in our cohort was high, mostly of moderate severity, and, typically, resulted in more co-medication. It is well-known that multimorbidity may influence patient decisions, favouring tolerability while trying to balance risks and potential benefits [6]. Recently published data has not reported significant impact on early treatment discontinuation of chemotherapy in cohort of 1213 patients with relapsed ovarian cancer [35]. Although only some physical and emotional domains of quality of life were described most salient, there is no prospective evaluation in ovarian cancer patients and distinct domains are more than heterogeneous [36, 37]. Exemplarily patients after extensive tumour debulking with gross bowel resection, who are not able to resorb oral drugs due to consequently insufficient bowel metabolism, needs quite different treatment strategy as an elderly and frail patient with accumulated gastrointestinal toxicity [35, 38]. Thus, knowledge of late effects of cancer survivals and their individual preferences could help to modify possible ineffective treatment and increase satisfaction with care, but have to be studied systematic in a prospective approach [13, 38].

Summarising elderly ovarian cancer patients demonstrated a high motivation to realize their treatment preference, despite their comorbidity, co-medication and previous chemotherapy experience. The preference for i.v. chemotherapy in this palliative cohort could be described with subjective expectations and individual explanations to toxicity, safety and treatment potency which are difficult to be quantified objective. As expected there were no severe toxicities or differences in efficacy observed [39, 40]. Thus the concept of a patient’s free choice following preference for the drug application form could be an attractive option in the treatment of ROC, especially in elderly and comorbid patients with heavily pre-treated recurrence.

## Conclusions

Elderly patients with recurrent ovarian cancer have clear preferences and are motivated to participate to the treatment decision process. In the palliative situation they preferred the i.v. application of treosulfan, based on individual experience with toxicity, comorbidity and co-medication, which reflects their specific geriatric situation.

## Abbreviations

ADL: Activities of daily living
AE: Adverse event/s
ECOG: Eastern Cooperative Oncology Group
iADL: Instrumental activities of daily living
ROC: Recurrent ovarian cancer
